# Enhanced education for adult patients with persistent post-concussion headaches: a randomized controlled trial

**DOI:** 10.2217/cnc-2022-0008

**Published:** 2023-01-04

**Authors:** Emily Collett, Tianru Wang, Candice Todd, Anil Dosaj, Andrew Baker, Cindy Hunt

**Affiliations:** 1 Dalla Lana School of Public Health, University of Toronto, Toronto, M5T 3M7, Ontario, Canada; 2 Division of Neurology, University of Toronto, Toronto, M5S 3H2, Ontario, Canada; 3 Head Injury Clinic, St. Michael's Hospital, Unity Health, Toronto, M5B 1W8, Ontario, Canada; 4 Concussion Ontario Network, Ontario Brain Institute, Toronto, M5H 3W4, Ontario, Canada; 5 Department of Anesthesia & Surgery, University of Toronto, Toronto, M5G 1E2, Ontario, Canada

**Keywords:** brain concussion, chronic post-concussive syndrome, patient education, randomized controlled trial, rehabilitation

## Abstract

**Aim & Patients:**

We conducted a randomized clinical trial to determine if an e-learning intervention can enhance recovery in adult patients with persistent post-concussion headaches (PPCH).

**Materials & Methods:**

The intervention consisted of three e-learning modules administered at baseline, 6 and 12 weeks. Data were collected on symptoms, community integration, quality of life and healthcare utilization at baseline and 12-week follow-up. ANCOVA was conducted to compare changes.

**Results:**

No statistically significant difference was observed on symptoms although we observed a trend toward reduced healthcare utilization and improved quality of life in the intervention group.

**Conclusion:**

E-learning modules for patients experiencing PPCH warrant further investigation with data on participant compliance and measures focusing on simpler short-term outcomes.

**Clinical Trial Registration**: NCT03391583 (ClinicalTrials.gov)

Concussion is defined as a physiological disruption of brain function and is manifested by loss of memory, altered mental status or focal neurological deficit(s), based on how the American Congress of Rehabilitation Medicine defined mild traumatic brain injury [1]. It is a common type of injury that is of public health significance. Langer *et al.* estimate the mean incidence of concussion in Ontario at 1153 per 100,000 residents, annually. The majority of individuals with non-sport-related concussions have their symptoms subside and return to normal functioning within 3 months [2]. However, up to 33% of individuals continue to suffer post-concussive symptoms months to years after injury [2–4], with a higher proportion of adult females being affected than males [5]. Among the post-concussive symptoms, headache is the most frequent symptom, with a prevalence as high as 47–95% [6]. Patients suffering from persistent post-concussion headache (PPCH) find it difficult to work in environments with physical labour, bright lights or read from computer screens [4,7]. PPCH may lead patients to avoid social situations as these can exacerbate symptoms [4,7]. The negative impact of PPCH is far reaching, going beyond patient quality of life to impact work force economic productivity [4,7].

Patients experiencing post-concussive symptoms can pose a significant cost on the healthcare system [3,8]. The healthcare services utilized by adults with post-concussive symptoms before receiving tertiary care comprise frequent emergency department, family doctor and walk-in clinic visits [3]. Individuals may incur further personal expenses with the utilization of chiropractic, massage, rehabilitation programs and other alternative therapies which are paid out of pocket and may be covered by insurance [3,9]. These services expire after a certain time [3,10], causing insurance coverage to be a limiting factor based on socioeconomic status. A minority of patients with concussions in Ontario experience post-concussive symptoms yet make up a large percentage of the overall direct healthcare cost to the healthcare system, with an average of $3000 per patient [3]. Treatment goals aim to reduce the frequency and/or severity of post-concussive symptoms in patients to shorten recovery time; reduce healthcare visits and costs; support return to work; and improve quality of life. Most studies investigating healthcare utilization after a concussion are observational. The randomized controlled trials (RCTs) that have studied the effects of education on healthcare utilization have mixed results. One trial, composed of veterans, found that participants who received the educational intervention addressing issues related to concussion and post-military deployment readjustment (e.g., stress, depression, pain and headache and returning to work) had lower odds of visiting the emergency department than participants receiving problem-solving therapy [11,12]. Whereas another study reported on the general population and did not find a statistically significant difference in healthcare visits between patients who received video information at discharge and those who received standard care [13].

Furthermore, there are mixed findings of the effects of educational intervention on treating patients with post-concussive symptoms. Mittenberg *et al.* conducted a RCT on adults with concussion using education via a printed manual, which provided information on the nature and course of expected symptoms, symptom reduction strategies and advice for gradual resumption of pre-injury activities [14]. At the 6-month follow-up, the intervention group had significantly fewer symptoms, lower symptom severity, and shorter symptom duration, compared with a standard care control [14]. Four other studies showed that similar educational interventions delivered verbally by clinicians [15–17] or through an information booklet [18], during concussion patients' acute recovery phase (i.e., 7–10 days post-injury), resulted in significantly lower symptom rates, less severe symptoms, shorter work absenteeism, and improved community integration [15–18]. Uncontrolled intervention and observational studies conducted on veterans and military service members with post-concussive symptoms indicated psychoeducational interventions (i.e., psychologist service with or without educational intervention) were effective in reducing symptom reporting during chronic recovery phase (i.e., ≥3-month post-injury) [19,20]. In contrast, two other educational interventions, one delivered verbally by an occupational therapist [21] and the other web-based [22], were not effective in treating post-concussive symptoms. Similar negative findings were also reported by one Cochrane review [23] and one systematic review [24]. The mode of delivery, length of time, number of sessions and the patient population differed slightly in these intervention studies, which could have resulted in the mixed findings. However, most interventions were only conducted in patients with post-concussive symptoms during the acute recovery phase. With most of the literature focusing on post-concussive symptoms; our study may fill in some of the knowledge gaps in the field regarding the effectiveness of education for symptom reduction in patients with PPCH during chronic recovery phase. Although the standard of care provides patients with headache counseling included in their assessments, patients often reported being overwhelmed by their visit to the Head Injury Clinic and that they were not able to retain self-care information shared by the clinical team. Therefore, the e-learning intervention has the potential to increase patients' knowledge on headache self-management techniques as they can access the information on their own time and as frequently as they would like. This intervention also allows patient's family/significant others to participate in their care by having access to the material.

The aim of this study was to compare the effects of an adjunctive e-learning educational intervention plus standard of care in a sample of adult patients with PPCH (chronic recovery phase) attending a tertiary care clinic. We hypothesized that three sessions of online educational intervention, delivered over 12 weeks, would result in significantly lower frequency and duration of symptoms, less healthcare service utilization, better community engagement, and higher quality of life in the intervention group compared with the controls.

## Materials & methods

### Trial design

This study is an RCT with parallel group design and an allocation ratio of 1:1, matched by sex (ClinicalTrials.gov Identifier: NCT03391583) [5,25].

### Participants

This study recruited participants who attended a tertiary care head injury clinic, over a 16-month timeframe. The inclusion criteria were: 18–65 years of age; a medical diagnosis of one or more concussion(s) determined by their family practitioner and a referral to the tertiary care clinic [26]; at least 3 months post-concussion; having persistent headaches and scoring 3 or more on the Rivermead Post-Concussion Symptoms Questionnaire (RPQ) at study baseline; being medically stable; comprehend English and could access email. Participants were excluded if they had a history of moderate to severe traumatic brain injury, other neurological (i.e., epilepsy, multiple sclerosis or Alzheimer's Disease), psychiatric or substance use disorders. Participants completed online surveys through REDCap at the baseline (upon entry to study) and 12-week follow-up. Demographic information (age and sex) was collected during enrolment, racial/ethnic-specific data were not collected because we needed to optimize our time with patients in the clinic.

### Intervention

Participants who met the eligibility criteria and gave consent were randomized to either the intervention group (received educational material and standard care) or the control group (received standard care only), with the standard of care being headache counselling. The current standard of care which participants in the control group received is not standardized in any formal matter as clinicians provide their own counselling. While many of the strategies featured in the intervention are discussed during appointments, patients may feel overwhelmed and anxious, potentially leading to poor information retention. Participants in the intervention group were provided with three additional PowerPoint slideshows developed by a neurology resident, adapted from material developed by the 3rd edition for clinical guidelines for concussion from the Ontario Neurotrauma Foundation [27]. The information included in the educational materials was determined according to the basic principles of acute and preventative therapies in headache medicine as well as examining prior research, other similar educational materials, and piloting the materials at the clinic prior to the beginning of the study. There were a priori learning objectives in the beginning of each lesson. The information in the modules could be found in public resources, as it was adapted from the clinical guidelines for concussion from the Ontario Neurotrauma Foundation [27]. However, the e-learning modules condensed relevant information from the clinical guidelines and translated it into summaries in plain language, tailoring to the specific population with PPCH in this study.

The first session was conducted in-person with a research assistant (RA) going through the first PowerPoint slideshow during the clinical visit and took approximately 30 min [28]. The topics included defining basic brain anatomy and post-traumatic headache, consequences of head trauma, common symptoms, approach to treatment, medications to avoid, when to seek medical guidance, and links to approved websites. The two remaining sessions of PowerPoint slideshows were emailed to the participants at the 6- and 12-week post-baseline timepoints for participants to self-direct and review at their leisure. Four RAs followed up by telephone to ensure the participants could open the email successfully. Session two recapped session one, explained the importance of keeping the brain active. Headache management techniques were also provided, including taking supplements (i.e., Vitamin D, Magnesium, Riboflavin, Co-enzyme), staying hydrated, exercising, caffeine-consumption, practicing mindfulness, avoiding alcohol and other substance use, and adopting healthy eating and sleeping habits [29]. The final session explained the influence of lifestyle on recovery, the role of stress and the importance of sleep and exercise on brain function and recovery [30]. Sessions two and three required approximately 10–20 min for the participants to view. To ensure treatment receipt and treatment fidelity, our research coordinator trained the RAs to use a checklist of criteria for which the delivery of the sessions should adhere. Furthermore, we attempted to track from the patient the amount of time spent on each education session, what was most/least helpful in each session. Participants allotted to the control group were offered the educational material at the end of the study. A $10 (Canadian) Tim Hortons gift card was given for each survey the participant completed.

This intervention was conducted in three separate sessions as to not overwhelm patients with too much information and limit screen time. It was delivered over 12 weeks within the time frame of their follow-up clinic appointments. This mode of delivery was chosen because it is easily accessible and allowed patients to review the information at their leisure. The current evidence around the effectiveness of technological devices as a health behavior change tool is limited and lacks sufficient details to know exactly which behavior change strategies delivered at what time points work the best [21–24]. The challenge is to find novel ways to extend healthcare by investing in novel services, using three sessions was decided by the clinical team experienced in care of post-concussion headache. The theoretical constructs of this intervention mode and their objectives are that self-management involves an individual managing their illness in a way that maximizes control over symptoms and quality of life [31]. Health services often provide strategies for self-management support to patients with chronic conditions. Interventions based on theories of behavior change focus on the provision of education to increase the patients' knowledge, skill, and confidence in managing their condition [31]. Techniques to change behaviours through implementation of technologies has been emphasized in the literature but to bridge the gap between theory and practice with patients attending tertiary care post-concussion has not been well studied. The shift from traditional face-to-face behavior change knowledge to learning from a digital platform presents a novel opportunity to our clinic. Developing the intervention by a trusted medical institution was a necessary precondition for patient engagement with the intervention. Within our clinical's scope of practice, patients travel from long and costly geographical distances, so we anticipated that the e-learning modules would significantly increase reach and access using an easy and low-cost platform to deliver these behavior change interventions.

### Outcomes

#### Primary outcome

Post-concussion symptoms, with a focus on headaches, were chosen as the primary outcome since headaches are often mismanaged and misdiagnosed in non-neurology practices, leading patients to use ineffective self-management strategies. The primary outcome of symptoms was measured by the RPQ, a 16-item questionnaire that evaluated the frequency and intensity of 16 common post-concussion symptoms compared with pre-injury [4]. Patients rate the frequency of symptoms on a Likert scale of 0 (‘Not experienced at all’) to 4 (‘A severe problem’). Responses of 1 (‘No more of a problem’) were considered a return to baseline frequency pre-injury and counted the same as a response of 0 [4]. All the answers were summed to calculate the total RPQ score. The first three items (headaches, dizziness, and nausea) were also summed to create RPQ-3 to measure the severity and frequency of headaches. The RPQ-3 was chosen as a measure of headache severity and frequency because the nausea and vomiting components of the score are common features of migraines and headaches in general [4].

#### Secondary outcomes

Healthcare utilization was chosen to investigate what healthcare services patients were using during their recovery and if service utilization decreased during the trial. This study also collected data on community engagement and quality of life to examine the ability of these patients to resume normal activities of life such as work and social relationships.

##### Healthcare utilization

Information was collected on participants' type and frequency of health service utilization in the past month at baseline and follow-up. Eight types of services were defined by the questionnaire: emergency department, family doctors, walk-in clinics, psychiatrists, neurologists, psychologists, rehabilitation (psychotherapist, occupational therapist, social worker, cognitive speech therapy), and alternative therapies (chiropractor, acupuncturist, osteopath, naturopath, massage therapy). This patient reported question, has been used in the clinic for several years by the clinical team and has been previously verified by comparing a sample of pre-clinic patient responses to clinic elicited responses [3].

##### Community engagement

Community engagement was measured by the Participation Assessment with Recombined Tools – Objective (PART-O), a 17-item scale that assessed overall participation over the three domains of productivity, social relations, and out and about [32]. The responses of each item range from 0 to 5, which corresponds to the lowest level of participation to the highest. The averaged total score was calculated by averaging the domain subscores [32].

##### Quality of life

Quality of life was measured by the Quality of Life after Brain Injury – Overall Scale (QOLIBRI-OS), a 6-item scale that assessed overall life satisfaction across six domains: physical condition, cognition, emotions, daily life, social relationships, and current situation and future prospects [33]. The responses of each item range from 1 (‘Not at all’) to 5 (‘Very’). The averaged total score was computed by summing up all items and dividing by the number of items answered (if no more than two responses were missing) [33]. The percentage scores were then calculated by subtracting 1 from the averaged total score and multiplying by 25, with higher scores indicating better perceived quality of life. The averaged total QOLIBRI-OS score was categorized into one of the four functioning categories ‘Impaired’, ‘Borderline’, ‘Normal’ or ‘Above average’ [33].

### Statistical analysis

A type 1 error at 5%, a power of 80%, and an anticipated attrition rate of 20% [4] was determined by the clinical team. Based on a review study by Theeler *et al.* [34] that showed a headache prevalence of 21-100% in the TBI group and 26% in the control group and according to the number of patients attending the clinic, this study aimed to enrol 35 participants in each study arm.

The participants were consented to the study from the tertiary care clinic and then randomly assigned to either group (allocation 1:1, matched by sex) by the RA using the computerized system randomize.net [35] with permuted blocks of random sizes. The block sizes were not disclosed to ensure concealment. The participants, as well as the clinicians were not aware of the allocation. The researchers who analysed the data were aware of the intervention assignment.

The descriptive statistics (i.e., mean, standard deviation, median, first and third quartile) of the outcomes were obtained. The Welch's *t*-test and Chi-squared test were conducted between the two groups on age, sex distribution and employment status, respectively. One-way analysis of covariance (ANCOVA) was performed, to compare the intervention group to the control group, on RPQ and RPQ-3, healthcare utilization visits, PART-O, and QOLIBRI-OS scores, using the baseline score as the adjusting covariate and the follow-up score as the dependent variable. All statistical analyses were conducted using SPSS for windows (version 27). p<0.05 was considered statistically significant.

### Research ethics

This study was approved by the St. Michael's Hospital Head Injury Clinic Steering Committee and the St. Michael's Hospital Research Ethics Board (Unity Health REB 17-227).

## Results

### Population demographics

**Figure 1.**
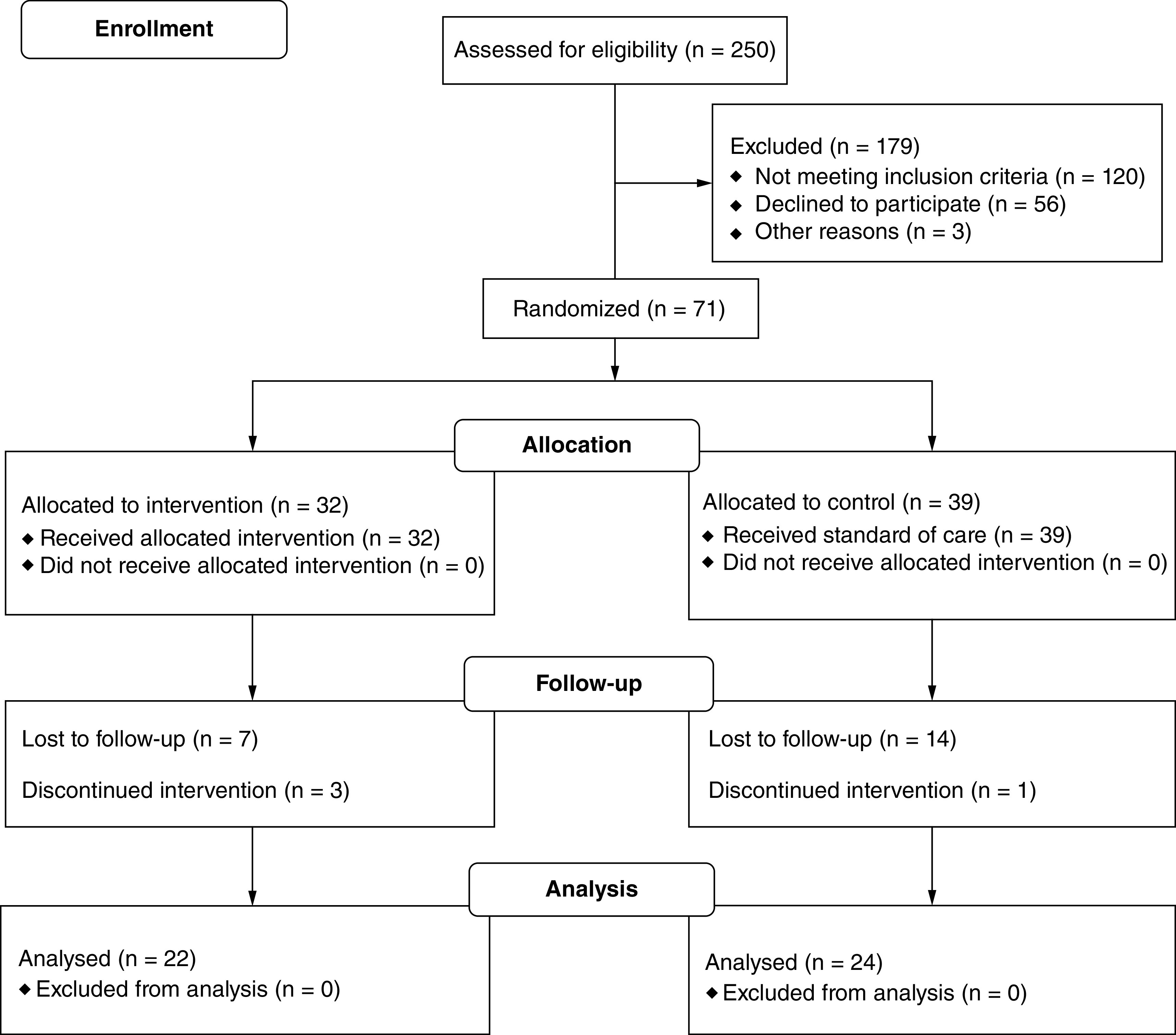
Flow chart of participants during enrolment, allocation and follow-up.

Figure 1 shows the flow of participants from enrolment until the end of follow-up. A total of 71 participants were enrolled in the study over a 16-month time frame. At baseline, 32 and 39 participants were randomized to the intervention and control group, respectively, with 22 participants in the intervention group and 24 participants in the control group completing follow-up study measures.

Table 1 displays the demographic information of the study population. At baseline, similar distributions were observed between the two groups' ages (mean age 45.13 [SD 12.99] years for the intervention group and mean age 41.57 [SD 12.42] years for the control group) and sex (71.88% intervention group and 71.79% of the control group were female). At follow-up, despite a similar sex distribution (77.27% intervention group and 79.17% control group were female; *χ*^2^ < 0.001, p = 1.000), participants that remained in the intervention group (mean age 47.10 [SD 12.37] years) were statistically significantly older than the control group (mean age 39.55 [SD 11.53] years; *t*_42.9_ = -2.137, p = 0.038). Most of the participants were employed at the time of follow-up (61.90% in the intervention group and 73.91% in the control group; *χ*^2^ = 0.276, p = 0.599).

**Table 1.** Participants' age, sex distribution and employment status.

	Intervention group		Control group		p-value
	n	Mean (SD)/%	n	Mean (SD)/%	
Age (years) at baseline^†^	32	45.13 (12.99)	39	41.57 (12.42)	0.246
Age (years) at follow-up	22	47.10 (12.37)	24	39.55 (11.53)	0.038
Sex distribution at baseline:					1.000
Male	9	28.13	11	28.21	
Female	23	71.88	28	71.79	
Sex distribution at follow-up:					1.000
Male	5	22.73	5	20.83	
Female	17	77.27	19	79.17	
Employment status at follow-up:					
Employed, paid full-time/part-time/self	13	61.90	17	73.91	0.599
Disabled, permanently or temporarily	6	28.57	3	13.04	0.219
Retired	1	4.76	0	0.00	0.965
Student	0	0.00	1	4.35	1.000
Temporarily laid off, sick/maternity leave	0	0.00	1	4.35	1.000
Other	1	4.76	1	4.35	1.000

† One participant in the intervention group and one in the control group did not choose any of the above options. Hence, the sample sizes sum up to 21 and 23 for the intervention and control groups, respectively (the percentages were calculated based on these numbers).

### Post-concussion symptoms

The median RPQ scores in the intervention group went from 33 to 32 while the scores in the control group remained constant at 26 over the study period. ANCOVA results showed no statistically significant differences in RPQ-3 (*F* = 0.258, p = .614) or RPQ scores between groups (*F* = 0.061, p = .805) (Table 2). Participants lost to follow-up had higher baseline median RPQ scores than participants who completed the study.

**Table 2.** ANCOVA results of healthcare utilization visits, and RPQ, PART-O, and QOLIBRI-OS scores.^†^^,^^‡^

	Intervention group (n = 22)				Control group (n = 24)				p-value
	Baseline		Follow-up		Baseline		Follow-up		
	Mean (SD)	Median (Q1–Q3)	Mean (SD)	Median (Q1–Q3)	Mean (SD)	Median (Q1–Q3)	Mean (SD)	Median (Q1–Q3)	
RPQ-3	6.1 (2.9)	5.0 (3.0–8.3)	5.6 (2.9)	6.0 (3.0–8.0)	5.8 (2.3)	6.0 (4.0–7.5)	5.0 (3.5)	5.0 (2.0–7.0)	0.614
RPQ	32.8 (14.3)	33.0 (21.0–43.8)	30.8 (16.3)	32.0 (15.5–42.8)	27.9 (15.2)	26.0 (16.0–37.0)	26.3 (18.2)	26.0 (10.5–39.8)	0.805
HU	16.9 (19.2)	7.0 (3.5–29.8)	5.1 (4.4)	3.0 (2.0–8.0)	11.5 (13.8)	5.0 (3.0–14.5)	4.9 (4.8)	3.0 (2.0–7.0)	0.946
PART-O	2.8 (0.4)	2.7 (2.5–3.0)	2.1 (0.7)	2.2 (1.7–2.7)	2.7 (0.4)	2.7 (2.3–3.1)	1.9 (0.7)	1.9 (1.3–2.6)	0.708
QOLIBRI-OS	37.5 (18.8)	37.5 (22.9–52.1)	38.7 (23.9)	41.7 (20.8–56.6)	41.2 (24.0)	41.7 (16.7–62.5)	36.8 (24.4)	37.5 (12.5–55.0)	0.320

† Participants lost to follow-up were excluded.

‡ Scores assessed were RPQ total score, and PART-O and QOLIBRI-OS averaged total score.

HU: Healthcare utilization visit; PART-O: Participation Assessment with Recombined Tools – Objective; QOLIBRI-OS: Quality of Life after Brain Injury – Overall Scale; RPQ: Rivermead post-concussion symptoms questionnaire.

### Healthcare utilization

The three most utilized health services in the intervention group were rehabilitation, alternative therapies (chiropractor, acupuncturist, osteopath, naturopath, massage therapy), and family doctors (Table 3). Rehabilitation visits in the intervention group went from 110 at baseline to 40 at follow-up, while visits in the control group went from 96 to 34. There was a total of 87 alternative therapy visits at baseline and 30 at follow-up in the intervention group and 78 visits at baseline and 29 at follow-up in the control group. There were 54 visits to a family doctor by participants in the intervention group at baseline and 16 visits at follow-up compared with 27 visits at baseline in the control group and 15 visits at follow-up. Neurologist visits changed from 12 at baseline to 2 at follow-up for the intervention group and from 15 to 12 in the control group. There were no visits to walk-in clinics in the intervention group from baseline through to follow-up and 19 visits at baseline and 2 at follow-up in the control group. The ANCOVA found no statistically significant differences in total healthcare visits (*F* = 0.005, p = 0.946) between the intervention and control groups from baseline to follow-up.

**Table 3.** Healthcare visits at baseline and follow-up.^†^

	Intervention (n = 16)			Control (n = 22)			p-value^‡^
	Baseline (%)	Follow-up (%)	Total (%)	Baseline (%)	Follow-up (%)	Total (%)	
ED	2 (0.7)	0 (0)	2 (0.5)	9 (3.6)	0 (0)	9 (2.5)	0.33
FD	54 (19.9)	16 (16.7)	70 (19.1)	27 (10.7)	15 (13.4)	42 (11.5)	0.22
Walk-in	0 (0)	0 (0)	0 (0)	19 (7.5)	2 (1.8)	21 (5.8)	0.65
Psychiatrist	2 (0.7)	4 (4.2)	6 (1.6)	6 (2.4)	7 (6.3)	13 (3.6)	0.30
Neurologist	12 (4.4)	2 (2.1)	14 (3.8)	15 (5.9)	12 (10.7)	27 (7.4)	0.16
Psychologist	4 (1.5)	4 (4.2)	8 (2.2)	3 (1.2)	13 (11.6)	16 (4.4)	0.82
Rehab	110 (40.6)	40 (41.7)	150 (40.9)	96 (37.9)	34 (30.4)	130 (35.6)	0.08
AT	87 (32.1)	30 (31.3)	117 (31.9)	78 (30.8)	29 (25.9)	107 (29.3)	0.14
Total	271	96	367	253	112	365	

† Participants lost to follow-up were excluded.

‡ ANCOVA results.

AT: Alternative therapy; ED: Emergency department; FD: Family doctor.

### Community engagement

The follow-up PART-O averaged total scores in both the intervention and control groups (2.1 vs 1.9) were lower than their pre-injury scores (2.8 vs 2.7 [Table 2]). There was no statistically significant change in the averaged total score (*F* = 0.144, p = 0.708) in the intervention group compared with the controls. At the 12-week time point, the intervention group did not have significantly greater community engagement compared with the controls.

### Quality of life

Over the study period, we observed a trend toward improved QOLIBRI-OS averaged total score in the intervention group (from 37.5 to 38.7) and a trend toward reduced QOLIBRI-OS in the control group (from 41.2 to 36.8 [Table 2]), yet no statistically significant change was found between the intervention group and controls (*F* = 1.013, p = 0.320) at follow-up. Regarding QOLIBRI-OS summary scores in the intervention group, 4 out of the 19 participants (21.05%) showed clinically significant improvements from a lower functioning category to a higher functioning category (i.e., impaired to normal), while 1 participant (5.26%) moved from a higher functioning category to lower. In the control group, only 1 of the 23 participants (4.35%) showed significant improvement from a lower functioning category to a higher functioning category, while 5 participants (21.74%) moved from a higher functioning category to lower.

### Harms

There were no harms to participants.

## Discussion

### Post-concussion symptoms

There was similar loss to follow-up within both groups (30%). Participants lost to follow-up had higher median RPQ scores than participants who completed the follow-up questionnaire. This finding may suggest that patients suffering from chronic headache find it challenging to read a screen for extended periods of time, thereby making study participation difficult to complete [4].

The intervention group did not have statistically significantly fewer symptoms as measured by the RPQ, including headaches post-intervention, compared with the controls. This may be partially explained by the mean age of the intervention group being significantly older than the control group, with the possibility that the intervention group might have had more age-related comorbidities and challenges with treatments, resulting in slower recovery [36]. In addition, the intervention group reported lower employment rates and higher disability rates than the control group. However, our study did not examine comorbidity or whether patients were involved with litigation or worker's compensation. Furthermore, we were unable to identify to what extent the participants in the e-learning condition read through the PowerPoints and took action (e.g., engaged in exercise, use a mindfulness strategy, took supplements), nor did we track activities in which those in the standard of care condition engaged in self-management strategies. This could potentially account for the lack of statistical differences between the two conditions on our primary outcome measures. Another possible reason for the limited statistical differences is that this study included patients with one or more concussions and people with multiple concussions may have more challenges in seeking resolution of their headaches.

Other RCTs examined the effects of an educational intervention on participants recruited from emergency departments [13,37,38]. One such study by Matuseviciene *et al.* [37], which consisted of providing patients with educational material, found no statistically significant differences in RPQ scores. Two other studies measured the effects of an educational intervention on RPQ scores [12,39]. Neither study, which followed participants for 12 months, had statistically significant differences in RPQ scores [12,39], which is consistent with our results.

### Healthcare utilization

There were no statistically significant differences in healthcare visits during the study period between the study groups. However, the result of the ANCOVA comparing rehabilitation visits between the intervention and control groups approached statistical significance (p = 0.08). This may indicate that the headache management techniques from the educational materials helped reduce rehabilitation service utilization as studies have shown that patients with more severe concussions utilize rehabilitation services at greater rates [40,41]. As rehabilitation, psychologist, and alternative therapy visits are either paid through insurance or out of pocket by patients, similar financial pressures would be exerted on both groups [3,40,41]. This may explain the overall changes observed in the intervention and control groups between baseline and follow-up for rehabilitation, psychologist and alternative therapy visits.

### Community engagement

We did not observe significantly increased community engagement in the intervention group compared with the controls (Table 2). This result is consistent with the findings of Bell *et al.* [42], in which the intervention group received a Centers for Disease Control (CDC) manual ‘Fact about Concussion and Brain Injury and Where to Get Help’, along with five scheduled telephone counseling. Two other RCTs conducted by Ghaffar *et al.* [21] and Elgmark, Andersson *et al.* [43] also suggest educational information provided by a rehabilitation specialist and occupational therapist, to patients with concussion, did not result in significantly different community engagement compared with the controls. Moreover, Paniak *et al.* [44,45] found patients who were provided with the National Head Injury Foundation's Minor Head Injury brochure and an education session, did not have significantly higher community integration scores, at 3-months or the 1-year, versus the controls. Several studies reported opposite findings. Wade *et al.* [46] found the intervention group that received an educational information leaflet in an emergency department and care from a specialist team (if needed) had significantly less disruption in social disability than the controls. Hinkle *et al.* [47] also had similar findings, whereby patients who received information verbally from clinicians on the expected symptoms at discharge, returned to social activities significantly sooner than the controls.

### Quality of life

Despite not finding significant increases in QOLIBRI-OS scores over the study period, we observed a trend toward improved quality of life in the intervention group versus the controls. This finding is consistent with Bell *et al.* [42], who showed no significant differences in quality of life between the intervention and control groups. Their study measured Modified Perceived Quality of Life (PQOL) at 6-month follow-up [42]. In contrast, Elgmark Andersson *et al.* [43] found statistically significant improvement in physical health and leisure as measured by Life Satisfaction Questionnaire (LiSat-11) at 1-year follow-up. Our study (n = 46) and Bell *et al.* (n = 311) [42] did not observe any significant improvement when participants were followed-up for 3 and 6 months, respectively, but Elgmark Andersson *et al.* (n = 355) [43] did when the 1-year follow-up was completed. Therefore, it is possible that a more evident improvement in quality of life will be observed during a longer study duration.

### Limitations

Our study findings are not generalizable to all concussion patients for several reasons. The number loss to follow-up was higher than expected and may have contributed to the null findings of the study [48]. Participants were recruited from a tertiary care clinic and they might have had more severe and chronic symptoms compared with participants recruited from a different environment. Time post-injury was not collected at baseline. As patients with a longer time post injury may have responded differently to the education than those with a more recent injury, the collection of this variable should be considered in a future analysis with a larger sample size. Compliance for the second and third sessions, which were delivered by email, was not assessed. In addition, learning and comprehension of the content from the educational materials were not measured. More time should be given between the receipt of the third session and the final follow-up to accurately ascertain the effects of the third intervention session on PPCH and to ensure participants have had enough time to read the materials. Data collected in this study was self-reported by patients, which might be more subjective than psychosocial interviews conducted with clinicians. Additional factors that we did not study that may have influenced the outcomes are comorbidities (e.g., serious mental illnesses, acute suicidality), medication use for current medical issues or PPCH, and social determinants of health such as education level, family supports and income. In the authors' opinion, many persistent concussion patients are likely to have undiagnosed migraine. The piece that is missing from the migraine criteria is photo and photophobia. Ideally, if we could go back, we would have included those pieces. A further consideration was that much of the educational materials addressed daily lifestyle management techniques, which might not be enough to help with chronic symptom management within this vulnerable patient population. In addition, prior to participating in the study, participants may have encountered educational materials outside of the tertiary care clinic that were similar to the materials given to the intervention group which may have affected their responses. It is possible that in our efforts to measure the effectiveness of the education program that we underestimated or missed the information that was in fact learned, by seeking to focus on whether symptoms improved. In fact, one could argue that our measures of symptom severity, healthcare utilization, societal participation, and quality of life, are all longer-range outcomes that require much more complex interventions. Whereas future studies may focus on measuring knowledge, skill use, or confidence [49]. Another limitation of the study was not including age as a blocking variable is randomization. Further ANCOVA analysis could explore if results change based on age. However, a larger sample size would be required to address this investigation. We also did not consider time since injury, which may play a role in outcomes as patients may be more motivated closer to the time of injury. Acceptance and adoption are concepts that relate to the decision to start using a new technology, however, patients need to stick with the technology and use it in the right way to really benefit [50]. Patients' motivation to use the e-learning modules could embed new headache self-management habits [50]. Moreover, family members were able to view the intervention and support the patient in self-management.

## Conclusion

This RCT compared outcomes of symptoms, community engagement, quality of life, and healthcare utilization in the intervention group that received three sessions of enhanced online education for PPCH and standard care, to the control group that received standard care. Our findings support several positive trends observed with the e-learning modules among patients experiencing chronic PPCH which included a reduction in healthcare utilization and improvement in quality of life.

All measures of patient reported data in this study were internationally recognized common data elements by the National Institute of Neurological Disorders and Stroke (NINDS) [51], to promote comparison of these findings with other studies internationally. With the measures we used and the sample we obtained, no statistically significant study outcomes were observed between the intervention and control groups.

With the high attrition rate observed in both groups, in our clinical environment a larger sample size is needed for future studies to measure the impacts of enhanced educational interventions for post-concussion headaches. Collecting information on participants' social determinants of health may help to understand their influences on educational outcomes. Patient's knowledge and understanding of TBI and PPCH as well as their general health perceptions at baseline should be assessed in future studies. These thoughts and perceptions, which can be the result of previous experiences in the healthcare system or be culturally informed, may influence whether patients receiving the intervention experience an improvement in symptoms. Future studies should also consider collecting data on treatment receipt and fidelity and feedback from participants and their families on the challenges of using the educational material. Understanding the obstacles to using the educational materials may help improve its effectiveness. For example, paper copies of the questionnaires and educational materials can be mailed to participants with light or screen sensitivity, fewer more succinct surveys could be used, or educational materials with different color schemes.

Online material can be more easily accessed, stored and updated compared with printed manuals, and allows patients and supportive family members to review at their convenience.

**Summary points**

**Introduction**

- A randomized clinical trial conducted to determine if an e-learning intervention can enhance recovery in adult patients with persistent post-concussion headaches.
- Up to 33% of individuals continue to suffer persistent post-concussive symptoms, including headaches, months to years after injury.
- The aim of this study was to compare the effects of an e-learning educational intervention with standard care in a sample of adult patients with post-concussion headaches (chronic recovery phase).
- We hypothesized that three sessions of online educational intervention, delivered over 12 weeks, would result in significantly lower frequency and duration of symptoms, less healthcare service utilization, better community engagement, and higher quality of life in the intervention group compared to the controls.

**Materials & methods**

- Over a 16-month timeframe, participants with persistent post-concussive symptoms 3 months post-concussion were recruited from a tertiary care clinic.
- Participants in the intervention group were provided with three additional PowerPoint slideshows that provided a variety of headache management techniques compared to patients in the control group who received the current standard of care.
- Data was collected at baseline and 12 weeks on post-concussion symptoms (Rivermead Post Concussion Symptoms Questionnaire), healthcare utilization, community engagement (Participation Assessment with Recombined Tools – Objective), and quality of life (Quality of Life after Brain Injury – Overall Scale).
- One-way analysis of covariance (ANCOVA) was performed, to compare the intervention group to the control group using the baseline score as the adjusting covariate and the follow-up score as the dependent variable.

**Results**

- At baseline, 32 and 39 participants were randomized to the intervention and control group, respectively, with 22 participants in the intervention group and 24 participants in the control group completing follow-up study measures.
- Over the study period, we observed a trend toward improved QOLIBRI-OSaveraged total score in the intervention group and a trend toward reduced QOLIBRI-OS in the control group, yet no statistically significant change was found between the intervention group and controls at follow-up.
- No statistically significant difference was observed on the primary outcome (post-concussion symptoms) or any of the secondary outcomes (healthcare utilization, community engagement, and quality of life).

**Discussion**

- Our findings support several positive trends observed with thee-learning modules among patients experiencing chronic PPCH which included a reduction in healthcare utilization and improvement in quality of life.
- Future studies should have a larger sample size and consider collecting information on participant’s social determinants of health, time since injury, as well as data on treatment receipt and fidelity.
- E-learning modules for patients experiencing post-concussion headaches warrant further investigation with more data on participant compliance and feedback and measures that focus on simpler short-term outcomes.
